# Cytogenetic and agronomic characterization of intergeneric hybrids between *Saccharum* spp. hybrid and *Erianthus arundinaceus*

**DOI:** 10.1038/s41598-018-38316-6

**Published:** 2019-02-11

**Authors:** Babil Pachakkil, Yoshifumi Terajima, Nobuko Ohmido, Masumi Ebina, Shin Irei, Hisayoshi Hayashi, Hiroko Takagi

**Affiliations:** 1 Tropical Agriculture Research Front, Japan International Research Center for Agricultural Sciences, Ishigaki, 907-0002 Japan; 2 Graduate School of Human Development and Environment, Kobe University, Kobe, 657-8501 Japan; 3 Institute of Livestock and Grassland Science, National Agriculture and Food Research Organization, Nasushiobara, 329-2793 Japan; 4 Okinawa Prefectural Agricultural Research Center, Itoman, 901-0336 Japan; 5 University of Tsukuba, Tsukuba, 305-8572 Japan; 6 Present Address: Tokyo University of Agriculture, Tokyo, 156-8502 Japan

## Abstract

In sugarcane (*Saccharum* spp. hybrid) breeding, introgression of useful genes via intergeneric hybridization is a powerful strategy for improving the crop productivity. *Erianthus arundinaceus* shows great potential in terms of useful traits; however, little is known about the cytogenetic and agronomic characteristics of intergeneric hybrids between these two species. Here, we examine the cytogenetic and agronomic characteristics, and relationships between the two in intergeneric F_1_ hybrids between modern sugarcane cultivar and *E*. *arundinaceus* identified by amplification of 5S rDNA markers and morphological characteristics. The nuclear DNA content of the hybrids varied from 6.07 to 8.94 pg/2C, with intra-clonal variation in DNA content and 5S rDNA sites. Genomic *in situ* hybridization revealed 53 to 82 chromosomes in the hybrids, with 53 to 56 derived from sugarcane and 1 to 29 from *E*. *arundinaceus*. There were significant positive correlations between the number of *E*. *arundinaceus* chromosomes and dry matter yield, millable stalk weight, single stalk weight, and stalk diameter, but not sucrose content, reducing sugar content, sucrose/reducing sugar ratio or fiber content. This detailed information on intergeneric F_1_ hybrids between modern sugarcane cultivar and *E*. *arundinaceus* will contribute to effective utilization of *E*. *arundinaceus* in sugarcane breeding.

## Introduction

Sugarcane is an economically important crop with worldwide production of 1.9 billion tons^1^, accounting for two-thirds of the world’s sugar production and providing feedstocks for bio-energy production. Further improvement of this crop’s productivity will contribute to promoting food sustainability and energy security. Highly polyploid (2*n* = 100–130) modern sugarcane cultivars (*Saccharum* spp. hybrid) were derived via complex interspecific crosses between the sugar-producing species *S*. *officinarum* (2*n* = 10x = 80) and the wild species *S*. *spontaneum* (2*n* = 40–128). This interspecific hybridization, which was first performed a century ago, plays a major role in improving yield, disease resistance, and adaptability to abiotic stresses in sugarcane cultivars. However, only a limited number of parental materials have contributed to this interspecific hybridization, resulting in a narrow genetic base and limiting the scope for further improvements in sugarcane production^2,3^.

To broaden the genetic base of modern sugarcane cultivars, breeders have expanded their interest to intergeneric hybridization utilizing sugarcane-related genera belonging to the so-called ‘*Saccharum* complex’, which includes *Erianthus*, *Miscanthus*, *Sclerostachya*, and *Narenga*^4^. Of these, *Erianthus arundinaceus* (Rez.) Jeswiet shows considerable potential as breeding material due the high biomass productivity, superior ratoon ability, and exceptional adaptability to biotic and abiotic stresses of resulting sugarcane cultivars^5^.

Despite the importance of the *Erianthus* germplasm in sugarcane breeding, the large genetic distance between *Saccharum* and *Erianthus* results in cross-incompatibility^6–8^, a major constraint in generating intergeneric hybrids. Further constraints arise from the difficulty of distinguishing between genuine intergeneric hybrids and self-progeny. However, recent development of efficient molecular tools aimed at identification of intergeneric hybrids, such as PCR-based analysis of length polymorphisms in 5S rDNA^3^, simple sequence repeats (SSRs)^7^, amplified fragment length polymorphism (AFLP)^9^, and genomic slot blot hybridization^10^ have greatly facilitated intergeneric hybridization between *Saccharum* and *Erianthus*. In line with this, several reports have documented fertile intergeneric hybrids from crosses between *S*. *officinarum* (2n = 10x = 80), as the female parent, and *E*. *arundinaceus*^3,7,11–13^, as well as its back crossing populations^14^. However, crosses between modern sugarcane cultivars and *E*. *arundinaceus* have been less successful^13,15^. Approximately 15.0% to 27.5% of the modern sugarcane genome originates from *S*. *spontaneum*^16^, having a huge impact on the crop improvements. Successful intergeneric hybridization between modern sugarcane cultivars and *E*. *arundinaceus* could therefore help further these advances in sugarcane production.

Intergeneric hybridization is a challenging but powerful tool for broadening the genetic base in polyploid crop breeding, having resulted in new crops such as Triticale^17^ and Tritordeum^18^. Understanding chromosome behavior in intergeneric hybrids, such as chromosome elimination and unreduced gametes resulting from different coexisting chromosomes, is important for practical application in introgression programs. In intergeneric hybridization between wheat × pearl millet and oat × maize, partial hybrids with chromosomes derived from pearl millet or maize as well as a haploid set of wheat or oat chromosomes were respectively generated^19,20^. Unreduced gametes often occur during intergeneric hybridization^21^ and have been observed in *Saccharum* × *Narenga*, *Saccharum* × *Sclerostachya*, *Saccharum* × *Sorghum* intergeneric hybrids^22^. Genomic *in situ* hybridization (GISH) of intergeneric hybrids between *S*. *officinarum* and *E*. *arundinaceus* (2*n* = 10x = 60) revealed a chromosome transmission rate of ‘n + n’, with the number of *Erianthus* chromosomes transmitted to the F_1_ generation varying from 25 to 30^13,14,23^. Furthermore, BC_1_ populations obtained by crossing intergeneric F_1_ hybrids with modern sugarcane cultivars resulted in ‘2n + n’ chromosome transmission^14^, whereas BC_2_ and BC_3_ generations displayed ‘n + n’ chromosome transmission^14,24^. Chromosome recombination between *S*. *officinarum* and *E*. *arundinaceus* has also been reported in BC_1_^23,24^, BC_2_ and BC_3_ generations^24^. Although the cytogenetic characteristics of intergeneric hybrids between *S*. *officinarum* and *E*. *arundinaceus* have been extensively examined, there has been little progress in the characterization of intergeneric hybrids between modern sugarcane cultivars and *E*. *arundinaceus*.

In addition to cytogenetic characteristics, agronomic traits of intergeneric hybrids are also important in identifying useful and undesirable genes. In wheat × rye intergeneric hybridization, disease resistance genes from rye were introduced into wheat; however, the resulting breadmaking quality of the hybrids was inferior to that of wheat^25,26^. Various beneficial traits of *Erianthus* are thought to be introgressed into intergeneric hybrids, such as a high polyphenol content in the roots^15^, nematode resistance^27^ and drought tolerance^28^. However, information on the potentially valuable agronomic characteristics of intergeneric F_1_ populations between *Saccharum* and *E*. *arundinaceus* are limited, possibly due to the very low success rate of this hybridization. Moreover, the relationship between these cytogenetic and agronomic characteristics also remains unknown, further hindering the effective utilization of intergeneric hybrids in sugarcane breeding.

In this study, we report, for the first time, the cytogenetic and agronomic characteristics and relationships between the two in intergeneric F_1_ hybrids between modern sugarcane cultivar and *E*. *arundinaceus*. The detailed findings will contribute to effective application of *E*. *arundinaceus* as a potential gene source in sugarcane breeding.

## Results

### Generation and identification of intergeneric hybrids

Ninety-three seedlings were obtained from crosses conducted in 2007, 2008, and 2010 (Table 1). Hybrid seedlings were identified based on length polymorphisms of the 5S rDNA spacer in *Saccharum* and *Erianthus*^3^. The 5S rDNA band length is 280 bp in the genus *Saccharum* and 420 bp in the genus *Erianthus* (see Supplementary Fig. S1). PCR enabled amplification of the entire 5S rDNA unit, with genuine hybrids displaying both parental bands. Based on PCR analysis, one of five (20.0%) seedlings obtained in 2007 (J08-12), 17 of 54 (31.5%) obtained in 2008 (Series J09), and 21 of 34 (61.8%) obtained in 2010 (Series J11) were identified as genuine intergeneric hybrids. In total, 39 (41.9%) clones were therefore identified as intergeneric hybrids based on the 5S rDNA marker. Despite displaying no bands corresponding to the *Erianthus* 5S rDNA spacer, ‘J09-2’ and ‘J11-12’ were also identified as possible intergeneric hybrids because of their morphological appearance, which was very similar to the other hybrids. Overall, 41 intergeneric hybrids were therefore selected.

**Table 1:** Numbers of seedlings that germinated and genuine intergeneric hybrids identified based on 5S rDNA markers from intergeneric crosses between modern sugarcane cultivar and *E*. *arundinaceus*.

Year crossed	Year screened	Series	Female parent (Sugarcane)	Male parent (*E*. *arundinaceus*)	No. of seedlings	No. of hybrids	Percentage of hybrids
2007	2008	J08	NiF8	JIRCAS1	5	1	20.0
2008	2009	J09	NiF8	JW4	54	17	31.5
2010	2011	J11	NiF8	JW4	34	21	61.8
Total					93	39	41.9

### Intra-clonal variation in 5S rDNA sites in the intergeneric hybrids

Of the 41 intergeneric hybrids identified, a total of 32 showing acceptable growth were selected for detailed cytogenetic analysis and vegetative propagation. For each hybrid, 2–5 vegetatively propagated clones were individually sampled for 5S rDNA site analysis by PCR amplification. As a result, 5S rDNA sites originating from both parent species were clearly detected in 24 of these hybrids, with no detectable variation among the vegetatively propagated clones (no ‘intra-clonal variation’). Meanwhile, six hybrids exhibited ‘intra-clonal variation’, with some vegetatively propagated clones exhibiting 5S rDNA sites from both parents and others exhibiting sites from *Saccharum* only (see Supplementary Fig. S1), though they are vegetatively propagated from stalk cuttings of one hybrid.

Based on this ‘intra-clonal variation’ in 5S rDNA sites, the 32 intergeneric hybrids were classified into three groups (Table 2): Group A, which showed no intra-clonal variation in 5S rDNA sites, Group B, which showed intra-clonal variation in 5S rDNA sites, and Group C, which included ‘J09-2’ and ‘J11-12’, both of which contained *Saccharum* 5S rDNA sites only, but determined as intergeneric hybrids based on their morphological characteristics.

**Table 2:** Nuclear DNA contents of intergeneric hybrids between modern sugarcane cultivar and *E*. *arundinaceus*.

Group	Clone	Mean ± SD (pg/2C)	CV (%)	Min.	Max.	Range
Sugarcane cultivar	NiF8	11.18 ± 0.02	0.19	11.16	11.21	0.05
*E*. *arundinaceus*	JW4	7.50 ± 0.02	0.26	7.48	7.52	0.04
*E*. *arundinaceus*	JIRCAS 1	7.50 ± 0.02	0.21	7.48	7.52	0.04
Group A	J08-12	8.94 ± 0.04	0.39	8.94	9.01	0.07
	J11-19	8.63 ± 0.04	0.46	8.59	8.68	0.09
	J11-1	8.55 ± 0.08	0.92	8.47	8.65	0.18
	J09-1	8.53 ± 0.03	0.33	8.48	8.55	0.07
	J09-10	8.47 ± 0.05	0.60	8.42	8.54	0.12
	J09-4	8.44 ± 0.19	2.26	8.11	8.59	0.48
	J09-8	8.43 ± 0.11	1.29	8.25	8.52	0.27
	J09-5	8.40 ± 0.04	0.51	8.36	8.45	0.09
	J09-16	8.38 ± 0.11	1.29	8.27	8.52	0.25
	J11-9	8.30 ± 0.04	0.47	8.27	8.36	0.09
	J09-3	8.28 ± 0.28	3.42	7.93	8.56	0.63
	J09-6	8.12 ± 0.09	1.10	8.02	8.22	0.20
	J11-4	8.10 ± 0.12	1.54	7.95	8.26	0.31
	J11-5	8.10 ± 0.11	1.31	7.96	8.20	0.24
	J09-13	8.04 ± 0.15	1.88	7.83	8.23	0.40
	J11-15	8.01 ± 0.25	3.07	7.58	8.17	0.59
	J11-8	7.99 ± 0.15	1.84	7.80	8.17	0.37
	J11-6	7.93 ± 0.20	2.54	7.78	8.20	0.42
	J11-18	7.42 ± 0.22	2.94	7.90	8.46	0.56
	J11-3	7.41 ± 0.52	7.05	6.83	7.84	1.01
	J11-10	7.27 ± 0.06	0.77	7.23	7.31	0.08
	J09-14	7.16 ± 0.05	0.75	7.08	7.23	0.15
	J11-17	7.11 ± 0.04	0.63	7.08	7.14	0.06
	J09-15	7.01 ± 0.09	1.34	6.90	7.14	0.24
Group B	J11-13	7.72 ± 0.49	6.35	6.98	8.27	1.29
	J09-12	7.59 ± 0.52	6.91	7.06	8.07	1.01
	J11-21	7.54 ± 0.73	9.64	6.29	8.10	1.81
	J09-11	7.15 ± 1.13	15.73	6.49	8.45	1.96
	J11-14	6.85 ± 0.29	4.29	6.52	7.20	0.68
	J11-7	6.07 ± 0.32	5.33	5.72	6.51	0.79
Group C	J09-2	7.31 ± 0.38	5.25	6.81	7.66	0.85
	J11-12	6.72 ± 0.15	2.31	6.59	6.97	0.38

Group A, hybrids with no intra-clonal variation in 5S rDNA sites; Group B, hybrids with intra-clonal variation in 5S rDNA sites; Group C, hybrids with no *Erianthus* 5S rDNA sites but identified based on morphological characteristics; Max., maximum value; Min., minimum value; SD, standard deviation.

### Nuclear DNA contents of the intergeneric hybrids and parents

The nuclear DNA contents of the 32 intergeneric hybrids and their parents were determined by flow cytometry (Table 2). The nuclear DNA content of the female parent ‘NiF8’ (*Saccharum* spp. hybrid) was 11.18 pg/2C, while that of the male parents ‘JW4’ and ‘JIRCAS1’ (*E*. *arundinaceus*) was 7.50 pg/2C. The nuclear DNA contents of the intergeneric hybrids varied widely, with the highest content of 8.94 pg/2C in ‘J08-12’ and lowest content of 6.07 pg/2C in ‘J11-7’. Flow cytometric measurements among individual vegetative parent clones were very stable, with a coefficient of variation (CV) of less than 0.3%. Conversely, CV values of flow cytometric measurements among individual vegetatively propagated clones of the intergeneric hybrids ranged from 0.33 (J09-1) to 15.73% (J09-11), supporting the hypothesis that some of the 32 intergeneric hybrids exhibited intra-clonal variation in their nuclear DNA contents.

### Genomic composition of the intergeneric hybrids

**Figure 1:**
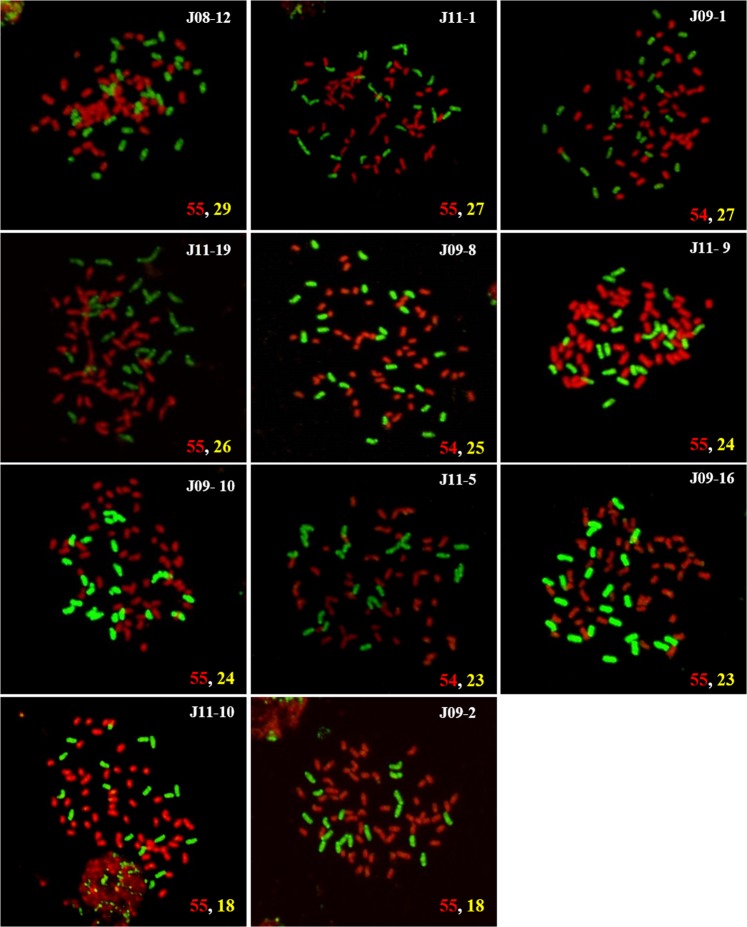
GISH analysis of intergeneric hybrids between modern sugarcane cultivar and *E*. *arundinaceus*. *Saccharum* chromosomes are shown in red and *E*. *arundinaceus* chromosomes in green. Numbers in the bottom right corner indicate the number of chromosomes from *Saccharum* (red) and *Erianthus* (yellow).

**Figure 2:**
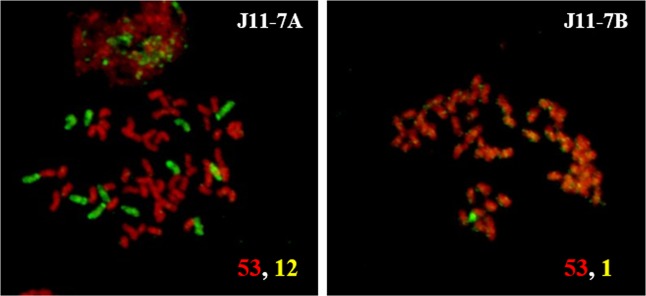
GISH analysis of vegetatively propagated clones of intergeneric hybrid J11-7. *Saccharum* chromosomes are shown in red and *Erianthus* chromosomes in green. Numbers in the bottom right corner indicate the number of chromosomes from *Saccharum* (red) and *Erianthus* (yellow).

**Figure 3:**
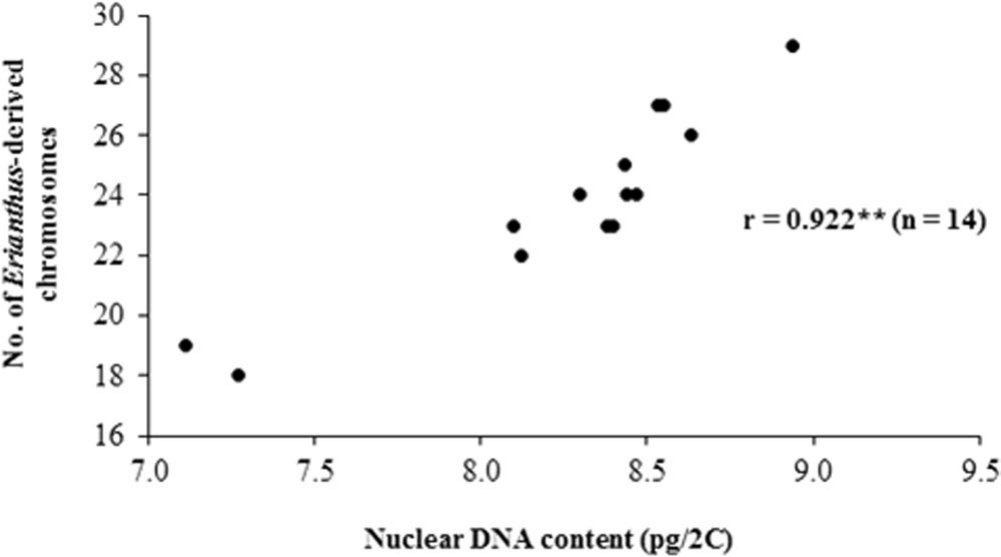
Correlation between the nuclear DNA content and number of chromosomes derived from *E*. *arundinaceus* in intergeneric hybrids from Group A.

To further determine the genomic composition of the intergeneric hybrids, 16 intergeneric hybrids were selected based on the variation in their nuclear DNA contents and 5S rDNA sites: 14 were from Group A, J11-7 from Group B, and J09-2 from Group C. The genomic compositions of these 16 intergeneric hybrids were determined by GISH. The mean chromosome number of the 14 intergeneric hybrids from Group A ranged from 71 to 82, with chromosomes derived from *Saccharum* and *Erianthus* ranging from 53 to 56 and 18 to 29, respectively (Table 3 and Fig. 1). GISH of two vegetatively propagated clones from buds on the same stalk of J11-7 (which exhibited intra-clonal variation in nuclear DNA content and the amplification of 5S rDNA sites from *Erianthus*) revealed 12 chromosomes from *Erianthus* in vegetative clone J11-7A, which was also found to contain *Erianthus* 5S rDNA sites and a nuclear DNA content of 6.6 pg/2C. Conversely, vegetative clone J11-7B, which contained no *Erianthus* 5S rDNA sites and had a nuclear DNA content of 5.7 pg/2C, contained only one *Erianthus-*derived chromosome (Fig. 2). ‘J09-2’ from Group C, which contained no *Erianthus* 5S rDNA sites, had a mean chromosome number of 72, with a mean number of chromosomes derived from *Saccharum* and *Erianthus* of 55 and 18, respectively (Fig. 1). There was also a significant positive correlation between the nuclear DNA contents of the Group A intergeneric hybrids and the number of chromosomes derived from *Erianthus* (Fig. 3).

**Table 3:** Chromosome composition of intergeneric hybrids between modern sugarcane cultivar and *E*. *arundinaceus*.

Group	Clone	Range of chromosome numbers observed (most common cytotype)		Total (Mean)	No. of cells observed
		From *Saccharum*	From *Erianthus*		
Group A	J08-12	52–56 (55)	26–29 (29)	79–85 (82)	20
	J11-1	52–56 (55)	26–28 (27)	79–84 (81)	19
	J09-1	53–55 (54)	25–28 (27)	78–83 (81)	35
	J11-19	53–56 (55)	24–29 (26)	78–85 (80)	17
	J09-8	52–55 (55)	23–26 (25)	76–81 (79)	20
	J11-9	54–56 (55)	22–26 (24)	76–81 (79)	11
	J09-4	54 (54)	24–25 (24)	78–79 (78)	3
	J09-10	52–55 (55)	22–24 (24)	74–79 (78)	18
	J11-5	54–56 (56)	20–24 (23)	75–80 (77)	19
	J09-5	52–54 (53)	23 (23)	75–77 (76)	14
	J09-16	52–55 (55)	22–26 (23)	75–81 (78)	16
	J09-6	53–56 (55)	22–25 (22)	75–80 (78)	7
	J11-17	52–57 (55)	16–19 (19)	70–77 (72)	16
	J11-10	51–56 (55)	16–19 (18)	68–75 (71)	23
Group B	J11-7A	52–54 (53)	12 (12)	64–66 (65)	5
	J11-7B	52–54 (53)	1 (1)	52–54 (53)	9
Group C	J09-2	53–56 (55)	16–18 (18)	70–74 (72)	14

Group A, hybrids with no intra-clonal variation in 5S rDNA sites; Group B, hybrids with intra-clonal variation in 5S rDNA sites; Group C, hybrids with no *Erianthus* 5S rDNA sites but identified based on morphological characteristics.

### Agronomic characteristics of the intergeneric hybrids

As shown in Table 4, there were significant differences (P < 0.001) and extensive variation between the hybrids with respect to all of the evaluated agronomic characteristics. The average dry matter yield of the hybrids was 591.0 g/stool, which was markedly lower than that of the female parent ‘NiF8’ (1621.9 g/stool) and the male parent ‘JW4’ (1419.3 g/stool). However, dry matter yield in the hybrids ranged from 40.3 to 1713.2 g/stool, with two hybrids (J08-12 and J11-1) showing even higher values than ‘NiF8’ (see Supplementary Fig. S2). Millable stalk weight ranged from 48.5 to 2446.9 g/stool, with a mean of 783.3 g/stool, which was lower than that of ‘NiF8’ (2766.7 g/stool) and ‘JW4’ (1165.6 g/stool). The mean number of stalks produced by the hybrids (10.8) was greater than that of ‘NiF8’ (6.4), but lower than that of ‘JW4’ (43.4) and the mid-parent value (24.9). The mean stalk length, stalk diameter, and single stalk weight of the hybrids were all lower than the corresponding values of ‘NiF8’ and the mid-parent value; however, the maximum hybrid stalk length was similar to that of ‘NiF8’. Conversely, the maximum stalk diameter and single stalk weight were lower than the corresponding values of ‘NiF8’ but much larger than those of ‘JW4’.

**Table 4:** Agronomic characteristics of intergeneric hybrids between modern sugarcane cultivar and *E*. *arundinaceus*.

Characteristic	NiF8	JW4	Mid- parent value	Intergeneric hybrids						
	Sugarcane (Female)	*Erianthus* (Male)		Mean (n = 32)	SD (n = 32)	Min (n = 32)	Max (n = 32)	h_2_ (n = 23)	CV_g_ (n = 23)	P (n = 23)
Dry matter yield (g/stool)	1621.9	1419.3	1520.6	591.0	512.0	40.3	1713.2	0.79	68.6	***
Millable stalk weight (g/stool)	2766.7	1165.6	1966.2	783.3	748.1	48.5	2446.9	0.73	74.7	***
Number of stalks (no./stool)	6.4	43.4	24.9	10.8	5.3	1.0	22.1	0.74	40.2	***
Stalk length (cm)	119.5	64.8	92.2	67.6	31.9	15.0	125.8	0.80	39.5	***
Stalk diameter (mm)	21.8	10.7	16.2	12.1	2.6	5.9	16.6	0.79	17.4	***
Single stalk weight (g)	949.8	262.0	605.9	252.9	169.5	51.2	644.1	0.91	57.9	***
Juice brix (%)	18.8	6.8	12.8	14.1	2.2	10.6	18.6	0.71	12.8	***
Sucrose (%)	17.8	3.1	10.4	8.5	2.8	2.3	18.0	0.52	20.4	***
Reducing sugar (%)	1.0	2.3	1.7	2.3	1.0	0.5	4.1	0.26	26.8	***
Sucrose/reducing sugar ratio (%)	94.9	56.5	75.7	77.6	11.4	49.6	95.6	0.46	10.6	***
Fiber (%)	10.2	23.4	16.8	16.7	3.3	8.0	22.4	0.64	15.1	***

Mean, SD, Min, and Max of the measured agronomic characteristics were calculated using data for the 32 hybrids tested. h_2_ and CV_g_ were obtained using data for the 23 hybrids for which three replicates were available.

Dry matter yield = total fresh weight × dry matter ratio.

Millable stalk weight = total fresh weight × millable stalk ratio.

Sucrose/reducing sugar ratio = (sucrose content/(sucrose content + reducing sugar content)) × 100.

Fiber content = (bagasse dry weight − (bagasse fresh weight − bagasse dry weight) × Juice brix% (100 −Juice brix%))/500 × 100.

CV_g_, genetic coefficient of variance; h_2_, heritability; Max, maximum value; Min, minimum value; SD, standard deviation.

^***^Significant difference at P < 0.001 among intergeneric hybrids.

The mean juice brix value of the hybrids (14.1%, range 10.6–18.6%) was comparable with the mid-parent value (12.8%), lower than that of ‘NiF8’ (18.8%) and higher than that of ‘JW4’ (6.8%). Similarly, the mean sucrose content (8.5%, range 2.3–18.0%) was comparable with the mid-parent value (10.4%), lower than that of ‘NiF8’ (17.8%) and higher than that of ‘JW4’ (3.1%). The mean reducing sugar content was remarkably higher than that of either parent, and the highest value observed was four times greater than that of ‘NiF8’. Meanwhile, the average sucrose/reducing sugar ratio was similar to the mid-parent value, but lower than that of ‘NiF8’ and higher than that of ‘JW4’. The average fiber content (16.7%, range 8.0–22.4%) was similar to the mid-parent value (16.8%), but higher than that of ‘NiF8’ (10.2%) and lower than that of ‘JW4’ (23.4%).

The broad-sense heritability of single stalk weight (0.91), stalk length (0.80), dry matter yield (0.79), and stalk diameter (0.79) of the hybrids was high, while that of the reducing sugar content (0.26) and sucrose/reducing sugar ratio (0.46) was low. The CVg of dry matter yield (68.6), millable stalk weight (74.7), and single stalk weight (57.9) was also high, while that of stalk diameter (17.4) and juice brix (12.8) was low.

### Relationship between cytogenetic and agronomic characteristics in the intergeneric hybrids

A significant positive correlation was observed between the number of *Erianthus* chromosomes in the intergeneric hybrids and dry matter yield, millable stalk weight, stalk diameter, and single stalk weight (Table 5). Intergeneric hybrids with *Erianthus* chromosome numbers close to the expected value of n = 30 showed vigorous growth, in contrast to those with fewer *Erianthus* chromosomes. However, an increased number of *Erianthus* chromosomes in the intergeneric hybrids was not correlated with the number of stalks, stalk length, juice brix, sucrose content, reducing sugar content, sucrose/reducing sugar ratio or fiber content.

**Table 5:** Correlations between agronomic characteristics and the number of *Erianthus* chromosomes in intergeneric hybrids from Group A (n = 14).

Character	Correlation coefficient
Dry matter yield	0.773**
Millable stalk weight	0.783**
Number of Stalks	0.336^ns^
Stalk length	0.457^ns^
Stalk diameter	0.697**
Single stalk weight	0.673**
Juice brix	−0.175^ns^
Sucrose	0.418^ns^
Reducing sugar	0.118^ns^
Sucrose / reducing sugar ratio	0.019^ns^
Fiber	−0.409^ns^

**Significance at a 1% level. ns: not significant at a 5% level.

## Discussion

Despite the potential of *E*. *arundinaceus* as germplasm aimed at broadening the genetic base and introducing new genes into sugarcane breeding, the cytogenetic and agronomic characteristics of resulting intergeneric hybrids have yet to be described in detail, and little is known about the relationships between these characteristics. This lack of data is therefore hindering the effective utilization of *E*. *arundinaceus*. To the best of our knowledge, this report is the first to investigate the cytogenetic and agronomic characteristics, and relationships between the two in intergeneric hybrids between modern sugarcane cultivar and *E*. *arundinaceus*.

### Generation of intergeneric hybrids between modern sugarcane cultivar and *E*. *arundinaceus*

Despite numerous attempts, very low rates of seed setting have been a major problem of intergeneric hybridization between modern sugarcane cultivars and *E*. *arundinaceus*. For example, no genuine hybrids were obtained between sugarcane cultivars and Indonesian *E*. *arundinaceus* ‘IK76-48’, ‘IK76-49’, ‘IK76-7’, or ‘IK 76-79’^13^. To the best of our knowledge, only Fukuhara *et al*.^15^ has so far reported successful intergeneric crosses between sugarcane cultivar ‘NiF8’ and Indonesian *E*. *arundinaceus* ‘IK76-126’ and ‘IJ76-349’; however, the percentage of genuine hybrids was very low at 2.4 and 2.3%, respectively. In this study, a total of 41 intergeneric hybrids were obtained from a cross between Japanese sugarcane cultivar and *E*. *arundinaceus* ‘JW4’, which is native to subtropical Japan, and ‘JIRCAS1’. Of these 41 hybrids, 39 were identified using 5S rDNA markers (Table 1) and two were identified morphologically, representing a total germination rate of 44.1%, which is successfully high compared with previous reports using Indonesian *E*. *arundinaceus* germplasm as the male parent^13,15^.

An important factor resulting in this successful hybridization rate is thought to be the genotypes of the parental material. Lee *et al*.^29^ revealed that the pollen tubes of Indonesian *E*. *arundinaceus* often fail to reach the micropyle of the *Saccharum* ovules during intergeneric hybridization, as also observed during intergeneric hybridization between wheat and pearl millet, whereby pollen germination and the pollen tube growth rate of male pearl millet on the wheat pistils varies between genotypes^20^. Genetic variation in *E*. *arundinaceus* is also affected by geographic landforms and local environments^30,31^, further that careful selection of the *E*. *arundinaceus* male parent could dramatically increase the success rate of intergeneric hybridization.

### Cytogenetic characterization of the intergeneric hybrids

In this study, the obtained intergeneric hybrids were classified into three groups based on variation in their nuclear DNA content and 5S rDNA sites: those without intra-clonal variation in 5S rDNA sites (Group A), those with intra-clonal variation in 5S rDNA sites (Group B), and those with *Saccharum* 5S rDNA sites only but characterized as intergeneric hybrids based on their morphological characteristics (Group C). GISH technique was subsequently used to determine the chromosome composition of the hybrids in Group A. The somatic chromosome numbers of sugarcane cultivar and *E*. *arundinaceus* are 2*n* = 110 and 2*n* = 60, respectively. GISH analysis of the 14 hybrids from Group A revealed that 54 to 56 chromosomes were transmitted from sugarcane cultivar, while 18 to 29 chromosomes were from *E*. *arundinaceus*. These results suggest that ‘n + n’ parental chromosome transmission occurred during hybridization, with varying degrees of *E*. *arundinaceus* chromosome elimination. Similar ‘n + n’ transmission of parental chromosomes was reported in intergeneric hybrids between *S*. *officinarum* and *E*. *arundinaceus*, with transference of 39 to 40 *S*. *officinarum* chromosomes and 25 to 30 *E*. *arundinaceus* chromosomes^3,13,14,23^. We observed no intergeneric hybrids from unreduced gametes between *Saccharum* and *E*. *arundinaceus*, in contrast to the unreduced gametes observed in intergeneric hybrids between *Saccharum* and *Sclerostachya*, *Narenga* and *Sorghum*^22^. DNA content analysis further confirmed that there were no unreduced gametes in the cross combinations between Japanese sugarcane cultivars and ‘JW4’ (data not shown). Moreover, no chromosome recombination between sugarcane cultivar and *E*. *arundinaceus* was observed in this study, although chromosome recombination between rye and wheat was previously identified during mitosis^32^. The elimination of *E*. *arundinaceus* chromosomes might occur more easily in intergeneric hybrids between modern sugarcane cultivar and *E*. *arundinaceus* compared with hybrids between *S*. *officinarum* and *E*. *arundinaceus*, possibly due to the large variation in *E*. *arundinaceus* chromosome number. As reported, modern sugarcane cultivars are derived from interspecific hybridization between *S*. *officinarum* and the wild species *S*. *spontaneum*, with approximately 15.0% to 27.5% of the modern sugarcane genome originating from *S*. *spontaneum*^16^. Lalitha *et al*.^33^ also reported elimination of *Erianthus* chromosomes in intergeneric hybrids between *S*. *spontaneum* and *E*. *arundinaceus*. The difficulties associated with intergeneric hybridization between modern sugarcane cultivars and *E*. *arundinaceus*, as well as the resulting variation in *E*. *arundinaceus* chromosome number, could therefore be attributed to the contribution of the *S*. *spontaneum* genome in modern sugarcane cultivars.

In hybrid ‘J11-7’ in Group B, intra-clonal variation was observed in *Erianthus* chromosome number between vegetatively propagated clones; that is, vegetative clone ‘J11-7A’ contained 12 *E*. *arundinaceus* chromosomes, while vegetative clone ‘J11-7B’ had only 1, even though they were vegetatively propagated from buds on the same stalk of J11-7 and separately maintained. In contrast, no intra-clonal variation in *Erianthus* chromosome number was reported in intergeneric hybridization with *S*. *officinarum*^3,13^. In other crops, chromosome or sequence elimination is a major response of wide hybridization^34^, with elimination tending to occur in the early stage of embryo development^35^. The intra-clonal variation in intergeneric hybrids between modern sugarcane cultivar and *E*. *arundinaceus* therefore suggests that *E*. *arundinaceus* chromosome elimination continued during the repetition of vegetative propagation as well as during embryo development.

Identification of genuine intergeneric hybrids based on 5S rDNA^3^ is highly efficient, and is therefore routinely used in sugarcane breeding programs around the world^15,27^. However, in this study, intergeneric hybrids belonging to Group C exhibited morphological characteristics similar to most hybrids in the remaining two Groups, despite lacking *Erianthus* 5S rDNA detection. In *Erianthus*, 5S rDNA sites are thought to be present on six chromosomes, with one site existing on one chromosome per set of basic chromosomes (x = 10)^3^. By performing fluorescence *in situ* hybridization analysis using the PCR product of the 5S rDNA as a probe, we were able to confirm 5S rDNA sites on six chromosomes in *E*. *arundinaceus* accession ‘JW4’. However, no 5S rDNA sites were detected in ‘J09-2’ in Group C (see Supplementary Fig. S3), suggesting elimination of all six *Erianthus* chromosomes carrying the 5S rDNA sites. Moreover, some hybrids in Group B also exhibited considerable inter-clonal variation among vegetatively propagated clones in terms of elimination of *E*. *arundinaceus* chromosomes and the PCR based detection of *Erianthus* 5S rDNA sites.

Based on the cytogenetic characteristics of the intergeneric hybrids between modern sugarcane cultivars and *E*. *arundinaceus*, attention should be given to chromosome elimination during vegetative propagation. In line with this, hybrids classified as Group A, which showed a stable *Erianthus* chromosome number during vegetative propagation, would be the most desirable breeding material for the generation of advanced backcross populations. The number of *E*. *arundinaceus* chromosomes in the intergeneric hybrids was also strongly correlated with the variation in DNA content revealed by flow cytometry (Fig. 3), suggesting the potential use of this technique as an efficient and simple tool for measuring the DNA content of intergeneric hybrids, which can otherwise be tedious and time consuming. This technique could also be useful in breeding programs during estimations of the number of *Erianthus* chromosomes transmitted to F_1_ hybrids. Overall, for identification of genuine intergeneric hybrids, it is better to combine 5S rDNA and other markers such as simple sequence repeats (SSRs)^9^.

### Variation in agronomic characteristics in the intergeneric hybrids in association with cytogenetic characteristics

Piperidis *et al*.^13^ previously suggested that intergeneric hybrids between *S*. *officinarum* and *E*. *arundinaceus* were very weak, with many failing to survive. In this study also, most of the intergeneric hybrids were relatively weak in terms of their agronomic characteristics, with a lower dry matter yield and lower millable stalk weight compared with their parents. The average stalk length, stalk diameter, and single stalk weight of the hybrids were also smaller than corresponding mid-parent values (Table 4). Although Roach (1978) reported heterosis in interspecific hybridization between modern sugarcane cultivars and *S*. *spontaneum*^36^, the intergeneric hybrids obtained here did not exhibit heterosis in yield-related traits.

The yield-related traits of intergeneric hybrids between sugarcane cultivars and related genera vary depending on the parent combinations. For example, intergeneric hybrids between sugarcane and *Sorghum*^37,38^, maize^39^, *Bambusa*^40^, and *E*. *procerus*^28^ resulted in hybrid weakness, while some hybrids between sugarcane and *Miscanthus*^41^ and *E*. *fulvus*^42^ showed improved growth compared to their parents. As suggested by molecular studies, *E*. *arundinaceus* is genetically more distant from *Saccharum* than *Miscanthus*, as is also true for *Sorghum* and Maize^8^. This large genetic difference therefore partly explains the hybrid weakness observed in hybrids between modern sugarcane cultivars and *E*. *arundinaceus*. Nevertheless, high CVg values were also observed for certain yield-related traits, such as dry matter yield, millable stalk weight, stalk number, stalk length, and single stalk weight, suggesting that selection and utilization of hybrids with desirable traits should be possible. In terms of quality-related traits, the female parent (modern sugarcane cultivar) is characterized by a high sucrose content, low reducing sugar content and low fiber content, while the male parent (*E*. *arundinaceus*) features a relatively low sucrose content, high reducing sugar content, and high fiber content. Meanwhile, their hybrids showed wide variation in these traits, although the average juice brix, sucrose content, and fiber content were comparable to the mid-parent values. Based on these observations, selection of hybrids with a relatively high sugar content, low fiber content, and low reducing sugar content for backcrossing could be possible.

As summarized in Table 5, significant correlations were observed between *E*. *arundinaceus* chromosome number and the values of certain agronomic characteristics. Yield-related traits such as dry matter yield, millable stalk weight, single stalk weight, and stalk diameter were positively correlated with *E*. *arundinaceus* chromosome number, with no correlation between *E*. *arundinaceus* chromosome number and sugar and fiber content (Table 5). In intergeneric hybrids between oat and maize, hybrids with more maize chromosomes were less vigorous than those with fewer^19^. In contrast, the hybrids obtained here showing unstable and fewer *Erianthus* chromosomes tended to be lower yielding, possibly due to incompatibility between the *Saccharum* and *Erianthus* chromosome sets. Further detailed analysis of the relationship between *Erianthus* chromosome number and agronomic characteristics in the intergeneric hybrids, including backcrossed populations, are therefore required.

### Application of the intergeneric hybrids in sugarcane breeding

The findings of this study suggest that intergeneric hybrids with an *E*. *arundinaceus* chromosome number of approximately n = 30 were more stable and showed good variation in sugar and fiber content. Such hybrids therefore show potential for utilization in sugarcane breeding. Since many intergeneric hybrids show weak vigor, evaluation and selection of hybrids for advanced backcrossing based solely on yield-related traits or high biomass production may be ineffective. Further efforts aimed at understanding the desirable characteristics of *E*. *arundinaceus* and developing phenotyping techniques are therefore required for effective application of resulting intergeneric hybrids in sugarcane breeding. Moreover, since the number of *Erianthus* chromosomes in the intergeneric hybrids decreased upon repeated backcrossing with modern sugarcane cultivars^14,23,24^ and since chromosome recombination between *S*. *officinarum* and *E*. *arundinaceus* has been reported in BC_1_^23,24^, BC_2_ and BC_3_ generations^24^, development of DNA marker-assisted selection techniques aimed at retaining desirable *Erianthus* chromosomes or fragments as well as eliminating those having an unfavorable effect are also required^14^.

Intergeneric hybrids reportedly exhibit low fertility or sterility, hindering backcrossing^13^. In our study, flowering was observed in 5 of the 41 hybrids, but all were sterile males. However, when a modern sugarcane cultivar was used as the male parent, successful production of BC_1_ and BC_2_ populations of intergeneric hybrid ‘J08-12’, which has 29 *Erianthus* chromosomes, was observed. Detailed evaluation of the agronomic and cytogenetic characteristics of these BC_1_ and BC_2_ populations are now ongoing. Nair *et al*.^28^ reported that intergeneric hybrids between *S*. *officinarum* and *E*. *procerus* had a low sucrose content, while BC_1_ progenies generated by crossing between the F_1_ and modern sugarcane cultivars had a significantly higher sucrose content. The intergeneric hybrids generated in this study tended to have a low sugar and high fiber content. Backcrossing with modern sugarcane cultivars is therefore also essential for utilization of these intergeneric hybrids in breeding programs aimed at retaining and improving sugar production. On the other hand, increasing demand for bagasse as an energy source is offering further incentive to develop new varieties showing increased fiber production. Further analysis of intergeneric hybrids with wide variation in fiber content could therefore help the balance between a high fiber content and reasonable sugar production.

## Materials and Methods

### Generation and identification of intergeneric hybrids

Intergeneric crosses were carried out at the Tropical Agriculture Research Front of the Japan International Research Center for Agricultural Sciences (JIRCAS-TARF), Ishigaki, Okinawa, from late November to December in 2007, 2008, and 2010 (Table 1). Commercial sugarcane cultivar ‘NiF8’ (progeny of the cross CP57-614 × F160) was used as the female parent, and *E*. *arundinaceus* accessions ‘JW4’ (2*n* = 60) and ‘JIRCAS1’ (2*n* = 60) were used as male parents. NiF8 is characterized by a high sugar content and disease resistance, and is used extensively in Japanese sugarcane production and breeding programs. Accession ‘JW4’, which was collected in Japan^30,43^ and is maintained in the JIRCAS-TARF germplasm collection, exhibits high biomass productivity in Japan’s temperate zones^44,45^. JIRCAS1 is also maintained in the JIRCAS-TARF germplasm collection, but its collection site is unknown. Intergeneric crosses were performed via biparental crossing using the standard procedures in JIRCAS-TARF, i.e. the marcotte and sulphurous acid solution method^46^, with emasculation of the female parent using hot water treatment (45 °C, 12 min). The ripened seeds were sown, and germinated seedlings grown in a greenhouse and verified for hybridization the following year.

Hybrid identification was conducted by PCR-based analysis of length polymorphisms of the 5S rDNA spacer regions in *Saccharum* and *Erianthus*. A Phire Plant Direct PCR Kit (Finnzymes, Finland) was used to extract genomic DNA from seedling leaf samples, and 5S ribosomal DNA (5S rDNA) primers [5′-TGGGAAGTCCT(C/T)GTGTTGC-3′ and 5′-(T/G)T(A/C)G(T/C)GCTGGTATGATCGCA-3′] were used for PCR amplification^3^. A final reaction volume of 25 μL was used, containing 2.5 mM MgCl_2_, 0.2 mM of each dNTP, 1 × Ex Taq Buffer (Takara Bio Inc., Kusatsu, Japan), 1 µM of each forward and reverse primer, 0.25 U Takara Ex Taq HS (Takara Bio Inc.), and approximately 20 ng template DNA. Reactions were performed using a GeneAmp PCR System 9700 thermal cycler (Applied Biosystems, Foster City, CA) with initial denaturation at 93 °C for 3 min and 30 s, followed by 35 cycles of 93 °C for 1 min, 55 °C for 40 s, and 72 °C for 1 min, and final extension at 72 °C for 5 min. PCR products were analyzed using 1% agarose gel electrophoresis and ethidium bromide staining. The identified intergeneric hybrids were multiplied carefully from the buds on the stalks of each hybrid (vegetatively propagated clones) and separately maintained in the fields to prevent mixing or contamination. Two to five plants per vegetative clone were analyzed to characterize intra-clonal variation at 5S rDNA sites in 2012.

### Estimation of nuclear DNA content

The nuclear DNA contents of the identified intergeneric hybrids and their parents (‘NiF8’, ‘JW4’ and ‘JIRCAS1’) were determined by flow cytometry using an Attune Acoustic Focusing Cytometer (Life Technologies, Carlsbad, CA). Fully developed young leaves were sampled in 2012 from the same vegetatively propagated clones used for PCR analysis. Parsley (*Petroselinum crispum*), which has a nuclear DNA content of 4.21 pg/2C, was used as an inter-reference. The nuclear DNA content of parsley was determined by flow cytometry using *Oryza sativa*, which has a nuclear DNA content of 0.90 pg/2C^47^, as an internal reference. Leaf blade samples (approximately 3 cm²) were finely chopped in 1.0 mL nuclear extraction buffer and their nuclear DNA contents were determined as described by Tsuruta *et al*.^30^. The DNA content of each sample was then calculated based on the amount of DNA in G1 stage nuclei relative to that for parsley. Flow cytometric analysis was performed two or three times with different leaf samples to confirm the DNA content of each intergeneric hybrid.

### GISH of the intergeneric hybrids

Based on their nuclear DNA contents, 16 intergeneric hybrids were selected for GISH. For chromosome preparation, root tips were excised from stalk cuttings placed in vermiculite, immediately pretreated with 0.1% 8-hydroxiquinoline for 3 h in the dark at room temperature then fixed in fresh fixative (ethanol:acetic acid 3:1). After fixation for 48 h, the root tips were transferred to 70% ethanol and preserved at −20 °C until use. Chromosome samples were prepared by the enzymatic maceration/air-drying (EMA) method^48,49^ with minor modifications, using an enzyme mixture consisting of 0.2% Onozuka RS cellulose and 0.1% pectolyase Y-23 adjusted to pH 4.2. The root tips were then macerated for 1 h at 37 °C. After air-drying, the macerated samples were observed with a light microscope, and preparations showing a sufficiently high proportion of metaphase cells were selected for GISH.

Genomic DNA extracted using the CTAB method^50^ from fully developed young leaves of parents ‘NiF8’ and ‘JW4’ was directly labeled using Nick translation mix (Roche, Basel, Switzerland) with the fluorochromes Cy3-dUTP (GE Healthcare, Buckinghamshire, UK) and digoxigenin-11-dUTP (Roche), respectively. GISH was performed according to the methods of Ohmido and Fukui^51^ with minor modifications. After hybridization, slides were washed three times at 42 °C for 5 min in 2 × SSC and for 5 min in 0.1 × SSC. Digoxigenin-labeled JW4 DNA was detected with antidigoxigenin-fluorescein (Roche). Slides were then washed three times for 5 min in 2 × SSC then three times in distilled water at room temperature. The slides were stained with DAPI and well-spread metaphase images were observed under a fluorescence microscope (Eclipse E800, Nikon, Tokyo, Japan) equipped with a sensitive cooled CCD camera (DS-Ri1, Nikon). Image capture was performed using blue, green, and ultraviolet excitation and emission filter sets.

### Evaluation of agronomic characteristics of the intergeneric hybrids

The evaluation of agronomic characteristics was conducted at JIRCAS-TARF (N 22 24′, E124 11) from April 2012 to February 2013. Soil in the experimental field was classified as Ultisol according to the USDA soil taxonomy^52^. Of the intergeneric hybrids generated, a total of 32 showed sufficient growth to provide planting material for the seedling nursery along with their parents, ‘NiF8’, ‘JW4’ and ‘JIRCAS1’. Stalk cuttings with one bud were planted in the nursery on 16 April 2012 and grown for nearly one month. The seedlings were then transplanted in the experimental field at 30 cm intervals with a row distance of 140 cm on 22 May 2012.

Sigmoidal-type slow-release fertilizer (LPS 120 type, 22 g N m^−2^, 8 g P_2_O_5_ m^−2^, 8 g K_2_O m^−2^) was applied on the day of planting. Five stools per plot (2.8 m^2^) with three replicates placed according to a randomized block design were prepared for 23 intergeneric hybrids and the parental varieties. Six hybrids were replicated twice and three only once due to difficulties with multiplication.

Plants were harvested between the 18 and 22 February 2013. To do so, 2^nd^, 3^rd^, and 4^th^ stools were harvested, and five stalks showing healthy growth were sampled after measuring the total fresh weight and stalk number per plot. Three of the five stalks were used to measure stalk length, stalk diameter (defined as the diameter of the minor-axis portion at the middle of stalk), single stalk weight, and the millable stalk ratio (defined as the summed weight of 3 millable stalks divided by the summed weight of 3 whole stalks). These stalks were then shredded and pressed using a cylinder press, forming well-mixed samples with a mass of 500 g each. Juice and bagasse samples were obtained from these mixed samples. Juice brix was measured using an electro-refractometer (RX-5000, ATAGO, Tokyo, Japan), and the sucrose, glucose, and fructose contents of the juice samples were determined by high performance liquid chromatography (Prominence, Shimadzu, Kyoto, Japan) with a RID-10A (Shimadzu) refractive index detector. Sugars were separated on a Shim-pack SCR101N column (Shimadzu) using distilled water as the mobile phase at a flow rate of 0.8 mL/min. The reducing sugar content was calculated as the sum of the glucose and fructose contents. The dry matter weight of the bagasse samples was measured after drying at 80 °C for 48 h. The remaining two stalk samples were chopped into small pieces and dried at 80 °C for 48 h to determine the dry matter ratio.

### Statistical analysis

The mean, standard deviation, and maximum and minimum values of the measured agronomic characteristics were calculated using data for the 32 hybrids tested. Analyses of variance, heritability (h_2_), and the genetic coefficient of variance (CV_g_) were performed using data for the 23 hybrids for which three replicates were available. Broad-sense heritability was calculated according to Burton and deVane^53^, Hanson^54^, and Thaikua *et al*.^55^, and was determined as σ^2^_G_/σ^2^_P_. Correlations between the number of *Erianthus* chromosomes in the intergeneric hybrids and observed agronomic characteristics were also analyzed using BellCurve for Excel (Social Survey Research Information Co., Ltd., Tokyo, Japan).

## Supplementary information

Dataset 1

## Data Availability

The datasets generated during and/or analyzed during the current study are available from the corresponding author on reasonable request.

## References

[1] Food and Agriculture Organization of the United Nations FAOSTAT Database. Rome, Italy, http://www.fao.org/faostat/en/#data (2018).

[2] Arceneaux G (1965). Cultivated sugarcanes of the world and their botanical derivation. Proc. Int. Soc. Sugar Cane Technol..

[3] D’Hont A (1995). Identification and characterisation of sugarcane intergeneric hybrids, *Saccharum officinarum *× *Erianthus arundinaceus*, with molecular markers and DNA *in situ* hybridisation. Theor. Appl. Genet..

[4] Mukherjee SK (1957). Origin and distribution of *Saccharum*. Bot. Gaz..

[5] Jackson, P. & Henry, R. J. Erianthus in *Wild crop relatives: Genomic and Breeding Resources*, Industrial Crops (ed. Kole, C.) 97–107 (Springer-Verlag, 2011).

[6] Alix K (1998). Isolation and characterization of a satellite DNA family in the *Saccharum* complex. Genome.

[7] Cai Q (2005). Verification of the introgression of *Erianthus arundinaceus* germplasm into sugarcane using molecular markers. Plant Breed..

[8] Sobral BW, Braga DP, LaHood ES, Keim P (1994). Phylogenetic analysis of chloroplast restriction enzyme site mutations in the *Saccharinae* Griseb. subtribe of the *Andropogoneae* Dumort. tribe. Theor. Appl. Genet..

[9] Aitken K (2006). Characterization of intergeneric hybrids of *Erianthus rockii* and *Saccharum* using molecular markers. Genet. Resour. Crop. Evol..

[10] Besse P, McIntyre CL, Burner DM, Almeida CG (1997). Using genomic slot blot hybridization to assess intergeneric *Saccharum *× *Erianthus* hybrids (Andropogoneae – Saccharinae). Genome.

[11] Krishnamurthi M, Sekar S, Rajeswari S, Kawar PG (2007). Introgression of *Erianthus* for the development of commercial sugarcane cultivars. Proc. Int. Soc. of Sugar Cane Technol..

[12] Nair NV (2006). Characterization of intergeneric hybrids of *Saccharum* using molecular markers. Genet. Resour. Crop. Evol..

[13] Piperidis G (2000). Molecular contribution to selection on intergeneric hybrids between sugarcane and the wild species *Erianthus arundinaceus*. Genome.

[14] Piperidis N (2010). GISH characterization of *Erianthus arundinaceus* chromosomes in three generations of sugarcane intergeneric hybrids. Genome.

[15] Fukuhara S (2012). Identification and characterization of intergeneric hybrid of commercial sugarcane (*Saccharum* spp. hybrid) and *Erianthus arundinaceus* (Retz.) Jeswiet. Euphytica.

[16] Piperidis G, Piperidis N, D’Hont A (2010). Molecular cytogenetic investigation of chromosome composition and transmission in sugarcane. Mol. Genet. Genomics.

[17] Wilson, A. S. On wheat and rye hybrids. *Trans*. *Proc*. *Bot*. *Soc*. *Edinburgh*. **12**, 286 (1876).

[18] Martin A, Chapman V (1977). A hybrid between *Hordeum chilense* and *Triticum aestivum*. Cereal Res. Commun..

[19] Riera-Lizarazu O, Rines HW, Phillips RL (1996). Cytological and molecular characterization of oat × maize partial hybrids. Theor. Appl. Genet..

[20] Ahmad F, Comeau A (1990). Wheat × pearl millet hybridization: consequence and potential. Euphytica.

[21] Liu D. *et al*. Distant hybridization: a tool for interspecific manipulation of chromosomes in *Alien gene transfer in crop plants*, *volume I: Innovations*, *methods and risk assessment* (eds Pratap, A. & Kumar, J.) 25–42 (Springer-Verlag, 2011).

[22] Kandasami PA (1961). Interspecific and intergeneric hybrids of *Saccharum spontaneum* L. I. Functioning of gametes. Cytologia.

[23] Wu J (2014). Unexpected inheritance pattern of *Erianthus arundinaceus* chromosomes in the intergeneric progeny between *Saccharum* spp. and *Erianthus arundinaceus*. PLoS One.

[24] Huang Y (2015). Characterization of chromosome inheritance of the intergeneric BC_2_ and BC_3_ Progeny between *Saccharum* spp. and *Erianthus arundinaceus*. PLoS One.

[25] Lowry JR, Sammons DJ, Baenziger PS, Moseman JG (1984). Identification and characterization of the gene conditioning powdery mildew resistance in ‘Amigo’ wheat. Crop Sci..

[26] Macri LJ, Ballance GM, Larter EN (1986). Factors affecting the breadmaking potential of four secondary hexaploid Triticales. Cereal Chem..

[27] Bhuiyan SA (2016). Assessment of resistance to root-lesion and root-knot nematodes in Australian hybrid clones of sugarcane and its wild relatives. Australasian Plant Pathol..

[28] Nair NV (2017). Characterization of an intergeneric hybrids of *Erianthus procerus *× *Saccharum officinarum* and its backcross progenies. Euphytica.

[29] Lee, D. J., Berding, N. & Bielig, L. M. *Saccharum* × *Erianthus* intergeneric breeding: pollination studies. *Proc. Aus. Soc. of Sugar Cane Technol.* **15**, 244–250 (1993).

[30] Tsuruta S (2017). Genetic variability in *Erianthus arundinaceus* accessions native to Japan based on nuclear DNA content and simple sequence repeat markers. Acta. Physiol. Plant..

[31] Zhang J (2017). How genetic variation is affected by geographic environments and ploidy level in *Erianthus arundinaceus*. PloS One.

[32] Xie Q (2013). Mitotic and meiotic behavior of rye chromosomes in wheat–Psathyrostachys huashanica amphiploid × triticale progeny. Genet. Mol. Res..

[33] Lalitha R, Premchandran MN (2007). Meiotic abnormalities in intergeneric hybrids between *Saccharum spontaneum* and *Erianthus arundinaceus* (Gramineae). Cytologia.

[34] Shaked H (2001). Sequence elimination and cytosine methylation are rapid and reproducible responses of the genome to wide hybridization and allopolyploidy in wheat. Plant Cell.

[35] Sanei M (2011). Loss of centromeric histone H_3_ (CENH_3_) from centromeres precedes uniparental chromosome elimination in interspecific barley hybrids. Proc. Natl. Acad. Sci. USA.

[36] Roach BT (1978). Utilisation of *Saccharum spontaneum* in sugarcane breeding. Proc. Int. Soc. of Sugar Cane Technol..

[37] Nair NV (1999). Production and cyto-morphological analysis of intergeneric hybrids of *Sorghum *× *Saccharum*. Euphytica.

[38] Thomas R (1930). Sugarcane-Sorghum hybrids. Agric. J. India.

[39] Janaki-Ammal EKA (1938). *Saccharum-Zea* cross. Nature.

[40] Rao JT, Alexander MP, Kandaswami PA (1967). Inter-generic hybridisation between *Saccharum* (sugarcane) and *Bambusa* (bamboo). J. Indian Bot. Soc..

[41] Chen WH, Huang YJ, Shen IS, Shih C (1986). Utilization of *Miscanthus* germplasm in sugar cane breeding in Taiwan. Proc. Int. Soc. of Sugar Cane Technol..

[42] Wang X-H (2010). Characterization of the chromosomal transmission of intergeneric hybrids of *Saccharum* spp. and *Erianthus fulvus* by genomic *in situ* hybridization. Crop Sci..

[43] Nagatomi S, Ohshiro Y, Nakasone S (1984). Expedition for sugarcane germplasm to the Ryukyu Islands: The first and second researches. Bull. Okinawa Agric. Exp. Sta..

[44] Ando S (2011). Overwintering ability and dry matter production of sugarcane hybrids and relatives in the Kanto region of Japan. JARQ.

[45] Matsunami H (2016). Effect of planting density and fertilizer application level on dry matter yield of *Erianthus arundinaceus* (Retz.) Jeswiet. Jpn. J. Grassl. Sci..

[46] Kinjo K (1994). Study on possible fertilization term of the sugar cane pistil under the method of marcotte sulphurous acid solution on sugar cane breeding. Bull. Okinawa Agric. Exp. Sta..

[47] Martínez SP, Arumuganathan K, Kikuchi H, Earle DE (1994). Nuclear DNA content of ten rice species as determined by flow cytometry. Genes Genet. Syst..

[48] Fukui K, Iijima K (1991). Somatic chromosome map of rice by imaging methods. Theor. Appl. Genet..

[49] Ha S (1999). Quantitative chromosome map of the polyploid *Saccharum spontaneum* by multicolor fluorescence *in situ* hybridization and imaging methods. Plant Mol. Biol..

[50] Fulton TM, Chunwongse J, Tanksley SD (1995). Microprep protocol for extraction of DNA from tomato and other herbaceous plants. Plant Mol. Biol. Reporter.

[51] Ohmido N, Fukui K (1995). Cytological studies of African cultivated rice. Oryza glaberrima. Theor. Appl. Genet..

[52] Hamazaki T (2005). Soil classification system in the world and application to agricultural and forestry: 5. Classification of cultivated soils in Japan, Third approximation: Outline and characteristics. Jpn. J. Soil. Sci. Plant. Nutr..

[53] Burton GW, de Vane EH (1953). Estimating heritability in tall fescue (*Festuca arundincea*) from replicated clonal material. Agron. J..

[54] Hanson, W. D. Heritability in *Statistical genetics and plant breeding* (eds Hanson, W. D. & Robinson, H. F.) 125–139 (National Academy of Science-National Research Council, 1963).

[55] Thaikua S (2015). Preliminary evaluation on digestibility and the relation to morphology and water content of *Brachiaria* spp. and their heritability. Grassl. Sci..

