# Supplementary material for: Cytogenetic and agronomic characterization of intergeneric hybrids between Saccharum spp. hybrid and Erianthus arundinaceus
Source: Sci Rep. 2019 Feb 11;9:1748. doi: 10.1038/s41598-018-38316-6 (PMC6370852; doi:10.1038/s41598-018-38316-6)
Supplement: Supplementary file 1 — Dataset 1 [file 41598_2018_38316_MOESM1_ESM.docx]

**Title of the manuscript:**

Cytogenetic and agronomic characterization of intergeneric hybrids between *Saccharum* spp. hybrid and *Erianthus arundinaceus*

**Author list:**

Babil Pachakkil, Yoshifumi Terajima, Nobuko Ohmido, Masumi Ebina, Shin Irei, Hisayoshi Hayashi and Hiroko Takagi


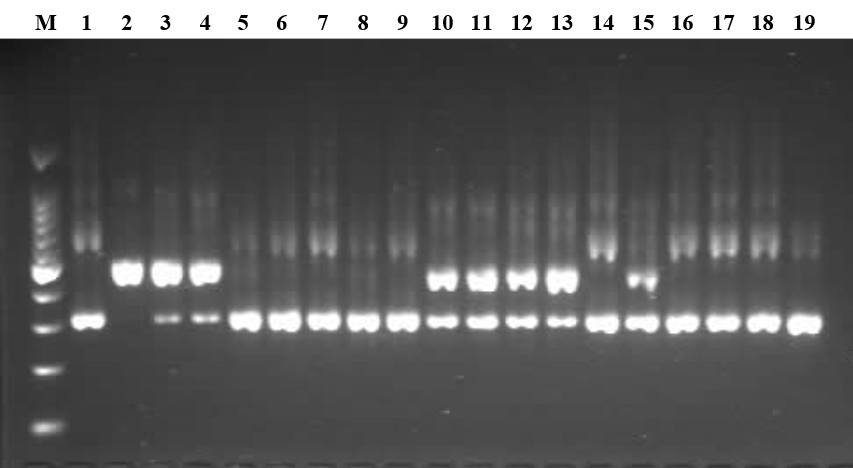


**Figure S1** Electrophoresis image of amplification of 5S rDNA sites on the modern sugarcane cultivar (Lane 1), *E. arundinaceus* (Lane 2) and intergeneric hybrids (Lane 3 to 19). 5S rDNA sites of both parents was detected in J11-10 (Lane 3 and 4). 5S rDNA sites of *E. arundinaceus* was not detected in J11-12 (Lane 5 to 9). Intra clonal variation was observed in intergeneric hybrids J11-13 (Lane 10 to 14) and J11-14 (Lane 15 to 19). Marker (Lane M) is 500 bp DNA ladder. This picture was taken by Yoshifumi Terajima.


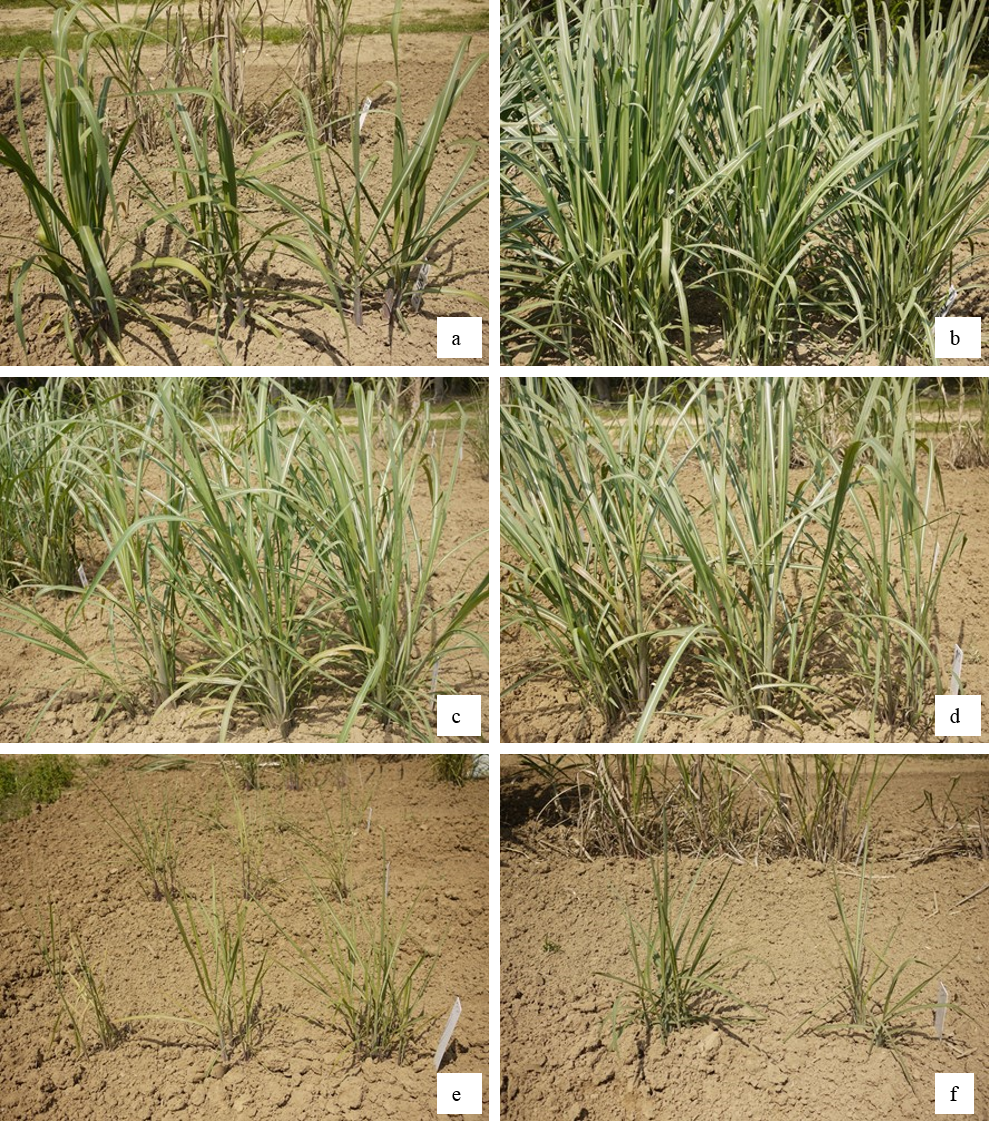


**Figure S2** Plants of intergeneric hybrids between modern sugarcane cultivar and *E. arundinaceus*. a: NiF8 (*Saccharum* spp. hybrid, female parent), b: JW4 (*E. arundinaceus*, male parent), c: J08-12 (intergeneric hybrid, Group A), d: J11-1 (intergeneric hybrid, Group A), e: J11-14 (intergeneric hybrid, Group B), f: J09-2 (intergeneric hybrid, Group C). These pictures were taken by Yoshifumi Terajima on 8 May 2013 in the ratooning field at JIRCAS-TARF.


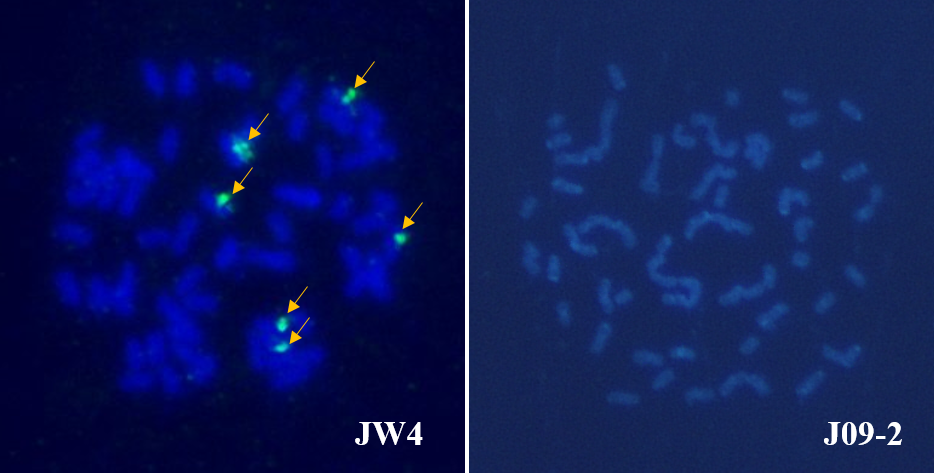


**Figure S3** Fluorescence *in situ* hybridization of *Erianthus* 5S rDNA sites on chromosomes of *E. arundin*aceus accession ‘JW4’ and intergeneric hybrid ‘J09-2’. The arrows on the chromosome image of ‘JW4’ indicate the locations of 5S rDNA sites. No 5S rDNA sites were detected in ‘J09-2’. We observed multiple cells to confirm the *Erianthus* 5S rDNA sites. These pictures were taken by Babil Pachakkil.
